# Leaders’ mental health in times of crisis: work intensification, emotional demands and the moderating role of organizational support and self-efficacy

**DOI:** 10.3389/fpsyg.2023.1122881

**Published:** 2023-05-02

**Authors:** Anja Wittmers, Günter W. Maier

**Affiliations:** ^1^Federal Institute for Occupational Safety and Health, Dortmund, Germany; ^2^Department of Psychology, Bielefeld University, Bielefeld, Germany

**Keywords:** COVID-19 crisis, leaders’ mental health, mixed methods, organizational resources, personal resources

## Abstract

This article focuses on leaders’ specific demands in times of crisis and the role of personal and organizational resources regarding mental health. The COVID-19 pandemic has led to increased levels of responsibilities, particularly among leaders. To deepen the understanding about the resulting consequences in terms of leaders’ demands and resources, we conducted a mixed methods study with a sample of 60 leaders from lower and middle management. We hypothesized leaders’ work intensification and emotional demands to be related with higher irritation and exhaustion. Consistent with the Job Demands-Resources model and the Conservation of Resources theory, we examined organizational instrumental support and occupational self-efficacy as possible moderators and assumed a buffering effect on mental illness. Our quantitative results indicated organizational instrumental support as a moderator for the relation of work intensification and mental illness. In terms of self-efficacy and work intensification, the results contradicted our expectations. For emotional demands, only the main effects could be found. In the qualitative part of our study, we found evidence for the importance of work intensification, emotional demands and organizational instrumental support in the leaders’ everyday experience and gained a deeper understanding of the constructs’ nature by means of examples. The integration of our quantitative and qualitative results has important and concrete implications for organizations how to support leaders in times of crisis and accelerated changes at work. This further underlines the necessity to consider leaders as an important target group of occupational health measures.

## 1. Introduction

In times of crisis a group, organization or community experiences a “serious threat to the basic structures or the fundamental values and norms of a system, which under time pressure and highly uncertain circumstances necessitates making vital decisions” (Rosenthal et al., 1989: 10). The COVID-19 pandemic caused a global health crisis and threatened the international economy. Besides the infection-related and economic costs, there is a vast range of social-psychological consequences for individuals in private and working life [Eurofound (European Foundation for the Improvement of Living and Working Conditions), 2020; Kniffin et al., 2020]. Regarding working life, the pandemic led to far-reaching changes within organizations (Robelski et al., 2020; Zacher and Rudolph, 2022) by addressing new demands such as infection prevention. Moreover, the pandemic was a catalyst for trends like digitization or mobile work that were already underway (Kniffin et al., 2020). Whenever a crisis occurs, its management is vital, a fact that highlights the salient role of leaders on various organizational levels. Given their high influence and decision latitude in organizations (e.g., Fischer et al., 2017), leaders are supposed to find suitable ways to actively manage the crisis (Boin et al., 2006; Zacher and Rudolph, 2022). Therefore, especially in uncertain times of crisis and change, followers need to be able to rely on positive and effective leadership behavior (Mumford et al., 2007; Hu et al., 2020; Klebe et al., 2021; Rudolph et al., 2021).

While leadership is a key determinant for followers´ health, performance, and organizational outcomes (Avolio et al., 2009; Skakon et al., 2010; Montano et al., 2017; Inceoglu et al., 2018), leadership research tends to neglect leaders’ own health (in this article we always refer to leaders’ mental health and psychosocial wellbeing) and their specific constellation of demands and resources (Zimber et al., 2015; Barling and Cloutier, 2017). This is a critical shortcoming as leaders´ health status has implications for their behavior toward followers (Byrne et al., 2014; Harms et al., 2017; Kaluza et al., 2020). While leaders are important for organizational functioning particularly in times of crisis, they are also confronted with crisis-related demands with likely consequences for their own health (Inceoglu et al., 2021). As Giustiniano et al. (2020, p. 971) summarized: Crisis leadership means, “guiding while being guided by contingencies.”

With our study, we aim to deepen the understanding of leaders’ specific demands in the course of the pandemic and the role of personal and organizational resources in relation to their health. Hence, we provide recommendations how to support leaders’ health in the COVID-19 crisis and beyond. We used a mixed methods design by combining quantitative and qualitative data to provide a more comprehensive view on our focal topic. The main target group of this study are operational leaders (i.e., leaders from lower and middle management, e.g., team leaders) who are sandwiched between economic requirements and organizational guidelines on the one hand and their followers’ needs on the other hand. There is empirical evidence that these kinds of leaders are facing more severe health risks than leaders on upper organizational levels (e.g., Björklund et al., 2013; Steidelmueller et al., 2020; Korman et al., 2022).

Specifically, we consider work intensification and emotional demands as two important challenges for leaders in times of crisis. Work intensification refers to an increase in terms of workload, variance of demands and time pressure (Kubicek et al., 2015; Paškvan and Kubicek, 2017). We regard it as a potentially salient stressor for leaders who are not only responsible for their own tasks but also for the functioning of a whole work unit. As a crisis implies alternation of work procedures (Mumford et al., 2007; Rascher, 2021), this responsibility might increase. Accordingly, leaders need to solve problems, address support requests or make decisions under time pressure and in ambiguous situations (Bluedorn et al., 1994; Hadley et al., 2011; Jungbauer and Wegge, 2015). Crisis-related emotional demands refer to the necessity of making and communicating difficult decisions with far-reaching consequences for individuals. Additionally, leaders have to deal with an increase of individual problems and worries among their followers (Mumford et al., 2007) – during the COVID-19 pandemic particularly due to the close link between private and working life (Kniffin et al., 2020).

In line with the Job Demands-Resources model (JD-R model, Demerouti et al., 2001), demands can have detrimental health effects. Due to the crisis and the necessity to handle new challenges, some of these demands are barely avoidable. Resources can have a buffering effect on the demand-health association (Bakker and Demerouti, 2017). Furthermore, according Conservation of Resources theory (COR theory, Hobfoll, 2001), the availability of resources corresponding to specific demands is particularly important. In the present study, we consider organizational instrumental support adapted to the COVID-19 situation and occupational self-efficacy as matching resources. Thus, we follow recommendations to integrate the organizational context as well as individual differences for the explanation of employees’ psychosocial reactions to the pandemic (Kniffin et al., 2020).

We contribute to the existing literature as follows: First, we add to the scarce but growing literature about leaders´ health. As leaders´ health has a strong influence on leadership behavior (e.g., Kaluza et al., 2020), and also a high relevance for followers and organizations, insights for health prevention of leaders are highly important. Second, we integrate the JD-R model and the COR theory to provide evidence for the buffering hypothesis of the JD-R model with a particular focus of the demands-resources match in times of crisis by underlining the importance of bespoke organizational and personal resources for leaders. Taking these contributions together, our study provides a constructive replication and extension (Köhler and Cortina, 2021) of previous findings with regards to work intensification, emotional demands, organizational instrumental support and occupational self-efficacy applied to a leaders and the crisis context (see also Venz and Boettcher, 2021). Third, based on our mixed methods approach, we advance the understanding of important demands and resources as well as of the buffering mechanisms of our focal resources in times of crisis. The qualitative dataset allows us to get deeper insights into the association between the constructs and to enrich the quantitative analysis. This is particularly helpful in terms of deducing concrete practical recommendations how to support leaders within organizations – in times of crisis and in times of accelerated change more generally.

## 2. Theoretical background

### 2.1. Job Demands-Resources model and Conservation of Resources theory as overarching frameworks

The JD-R model is conceptualized as an overarching model to explain health and motivation at work (Demerouti et al., 2001; Bakker and Demerouti, 2017). In particular, the model proposes health impairment as a consequence of demands, and motivation as a consequence of resources at work (for a detailed description of the dual process of the JD-R model see, e.g., Bakker and Demerouti, 2017). In this study, we focus on the interaction between leaders´ demands and resources depicted by the buffering tenet of the JD-R model (Bakker et al., 2005): Sufficient resources can mitigate the negative consequences of demands for health, whereas the combination of high demands and low resources implies a high risk of health impairment (Bakker et al., 2005). Looking into demand-resource mechanisms is highly relevant’ experiences and well-being in times of crisis-related changes. Particularly important are concrete changes in the employees´ work environment and the potential threat of resources and risks of lack of reciprocity in terms of resource investments (Halbesleben and Wheeler, 2011; Zacher and Rudolph, 2022). Both the threat of resource loss and the lack of success in re-investing resources in times of change and crisis can lead to impaired mental health (Hobfoll and Freedy, 1993). According to COR theory (Hobfoll, 1989, 2001; Hobfoll et al., 2018), individuals strive to obtain, retain, foster, and protect those things they centrally value, i.e., resources. COR theory underlines the potential effect of a crisis with the primacy of resource loss: Resource loss is disproportionately more salient than resource gain. Individuals invest their resources to deal with threatening conditions and prevent themselves from negative outcomes. Against the background of threatened or actual resource loss, resource gains are even more important (Hobfoll et al., 2018). Both JD-R model and COR theory assume the moderating role of resources. COR theory additionally states that a resource’s valance or, respectively, its match to the demands at hand improves its buffering potential (Hobfoll, 2001). Thus, resources should align to the specific needs of an occupational group. A lack of demand-resource fit explained inconsistent findings regarding the buffering hypothesis of the JD-R model in past empirical research (see Bakker and Demerouti, 2017).

### 2.2. Work intensification as a change-related demand

The static equivalent of work intensification is work intensity which is still one of the most salient and prevalent work stressors with a highly demanding character (Lohmann-Haislah, 2020) in terms of the JD-R model. Work intensification refers to an increased work intensity reflecting a dynamic aspect of work (Franke, 2015; Kubicek et al., 2015). COR theory underlines that such changes at work will be stressful due to increasing the risk of resource loss (Hobfoll and Shirom, 2001). It is a multi-faceted construct characterized by higher workload, increased fragmentation and reduced time for breaks (Franke, 2015; Kubicek et al., 2015). We expand this definition by two aspects: First, it will not only occur in terms of a quantitative increase, but also by getting new tasks and responsibilities without having the possibility to compensate for this qualitative increase of work (see, e.g., Zeytinoglu et al., 2007). Second, work intensification can lead to spillover effects with regard to individuals´ private life. Hence, working in leisure time as well as permanent availability should also be considered when trying to operationalize work intensification (Schulz-Dadaczynski, 2020).

As opposed to their followers, operational leaders are generally more often confronted with high demands as regards work intensity (Steidelmueller et al., 2020). In times of crisis, they are additionally confronted with the necessity to reorganize processes and procedures within their work unit (Rascher, 2021), e.g., in terms of leading virtual or hybrid teams. Moreover, they have a responsibility for occupational safety issues, which during COVID-19 crisis has become a significantly more prevalent demand. Critical situations can bring along the necessity of more directive leadership in terms of clear decisions, detailed directions, and structured tasks (Stoker et al., 2019). This is not only rooted in the organization’s pursuit of efficiency and control, but also in the followers’ need for orientation (Rast et al., 2013). The need for clear and rapid decisions – combined with that to adapt the way they lead (e.g., virtual leadership) – will probably enhance work intensification among leaders.

Empirically, previous studies could show that work intensification explains additional variance in health outcomes above the effects of the static concept of work intensity and other cognitive, emotional and physical demands (Kubicek et al., 2012; Franke, 2015). Work intensification has been examined with respect to stress (Zeytinoglu et al., 2007), burnout (Kubicek et al., 2012) or psychosomatic complaints (Franke, 2015).

Two relevant mental health outcomes of demanding working conditions are irritation and exhaustion. Irritation captures strain evoked by a perceived imbalance between resources and demands and has both cognitive and emotional components (Mohr et al., 2005). Irritation is a short-term mental health outcome and antecedes severe mental health impairment (Mohr et al., 2005). Exhaustion is defined as a consequence of intensive physical, affective, and cognitive strain and thus, is conceptualized as a longer-term consequence of exposure to certain job demands (Demerouti et al., 2003).

Against this background, we hypothesize:

*H1*: Work intensification will be positively related to (a) irritation and (b) exhaustion.

### 2.3. Crisis-related emotional demands

We suggest emotionally challenging situations to be another important demand during crisis. Emotional demands occur when individuals have to deal or are confronted with other people’s feelings at work (Burr et al., 2019). Times of crisis are particularly challenging for leaders with regard to emotional processes (Jungbauer and Wegge, 2015). The COVID-19 pandemic has led to psychosocial problems in private and working life [Eurofound (European Foundation for the Improvement of Living and Working Conditions), 2020; Klopprogge et al., 2020]. Particularly in private life, the diversity of problematic situations has increased, for example due to social isolation and work-privacy conflicts regarding elder or childcare duties. As private problems can affect workplace behavior and performance, leaders have – on top of all their other demands – to deal with their followers´ problems in this respect. Leaders are supposed to provide psychological resources such as feedback, support, and inspiration through regular contact with their followers (Kniffin et al., 2020). This can be problematic for their own health. Research has shown that supportive leadership behavior can deplete leader resources (Zwingmann et al., 2016; Kaluza et al., 2020). Furthermore, we suggest that making and communicating decisions under conditions of high uncertainty is another emotional demand for leaders in times of crisis (Grunberg et al., 2006). While leaders have to deal with those emotionally challenging situations, they are also due to regulate their emotions in order to signal stability and spread a positive mood which can be demanding for themselves. Demonstrating stability and fostering the unit’s mood is salient due to the risk of emotion and mood contagion from leaders to followers (Sy et al., 2005; Bono and Ilies, 2006) which is more probable and critical in crisis situations (Jungbauer and Wegge, 2015). Hence, we assume:

*H2*: Emotional demands will be positively related to (a) irritation and (b) exhaustion.

### 2.4. Organizational instrumental support as a buffering job resource

Organizational support refers to interventions undertaken by an organization to support their employees in difficult work situations (Eisenberger et al., 2020). Particularly, we differentiate between perceived organizational support and actually received support. We focus on actually received instrumental support by the organization. This kind of support becomes crucial when people are already confronted with demands as in an unexpected crisis (Kienle et al., 2006). In such a situation, organizations should consider providing immediate tangible resources (Kniffin et al., 2020). Instrumental support includes providing sufficient and timely informational as well as practical support (e.g., Veiel, 1985).

Informational support is closely connected to informational justice, which refers to the accessibility to information regarding organizational procedures (Colquitt, 2001). Times of crisis are times of insecurity, so-called weak situations (Shamir and Howell, 1999) with high ambiguity, when orientation through clear signals and information is highly important. Informational justice not only considers the access to information, but also whether this information is perceived as true and specific and is delivered in a transparent and timely manner (Colquitt, 2001; Maier et al., 2007). In this way, uncertainty can be avoided (Van den Bos and Lind, 2002; Burr et al., 2019). Operational leaders are responsible for communicating and justifying strategic organizational decisions within their teams. Therefore, being fully and timely informed about relevant changes and the effects for their teams is particularly important for leaders. In a comprehensive qualitative study about leaders´ challenges and needs in restructurings operational leaders reported that sufficient information and communication policies were decisive characteristics of an effective change and crisis management (Thomson et al., 2018).

In order to handle concrete practical challenges in times of crisis, informational support should go along with practical support. During the COVID-19 crisis, this included clear instructions for procedures and responsibilities particularly with regard to occupational safety and health (OSH) issues. These included OSH expert support, reliability of the technical infrastructure and access to training and information (e.g., Federal Institute for Occupational Safety and Health/BAuA, 2020; Federal Ministry of Labor and Social Affairs/BMAS, 2020).

This bundle of measures can give orientation and safety to operational leaders in terms of work intensification (e.g., by knowing how to reorganize processes and how to deal with occupational safety issues) and emotional demands (e.g., by getting sound arguments for difficult decisions and enough information to avoid unnecessary uncertainty within their teams). Accordingly, we hypothesize:

*H3*: Organizational instrumental support will moderate the positive relationship between work intensification and (a) irritation and (b) exhaustion. That is, the relation of work intensification with (a) irritation and (b) exhaustion will be weaker when organizational instrumental support is high (vs. low).

*H4*: Organizational instrumental support will moderate the positive relationship between emotional demands and (a) irritation and (b) exhaustion. That is, the relation of emotional demands with (a) irritation and (b) emotional exhaustion will be weaker when organizational instrumental support is high (vs. low).

### 2.5. Occupational self-efficacy as a buffering personal resource

Apart from the organizational context, individual differences are important for the explanation of employees’ psychosocial reactions to the pandemic (Kniffin et al., 2020). Therefore, we additionally consider personal resources in terms of occupational self-efficacy. Individuals with a high self-efficacy believe in their ability to control and master a threatening situation (Bandura, 1994). Occupational self-efficacy refers to the extent of one’s belief in one’s own ability to successfully complete tasks and reach goals in working life (Burr et al., 2019). Self-efficacy has been recognized by Hobfoll (2002) as crucial for individual adaptability, and, therefore, as decisive in changing conditions such as a crisis. Personal resources can buffer the undesirable impact of job demands on mental health (Bakker and Demerouti, 2017). In particular high levels of self-efficacy provide greater mastery to the individual, help to deal with demanding conditions and to prevent from negative health outcomes (Van Yperen and Snijders, 2000; Xanthopoulou et al., 2007; Bandura, 2009). Hence, experiencing occupational self-efficacy can be helpful for operational leaders to confidently deal with higher work intensity and emotional challenging situations.

Thus, we hypothesize:

*H5:* Occupational self-efficacy will moderate the positive relationship between work intensification and (a) irritation and (b) exhaustion. That is, the relation of work intensification with (a) irritation and (b) exhaustion will be weaker when occupational self-efficacy is high (vs. low).

*H6:* Occupational self-efficacy will moderate the positive relationship between emotional demands and (a) irritation and (b) exhaustion. That is, the relation of emotional demands with (a) irritation and (b) emotional exhaustion will be weaker when occupational self-efficacy is high (vs. low).

**Figure 1:**
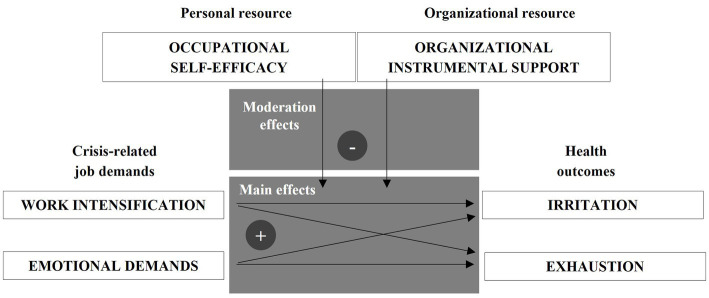
Research model.

The whole research model summarizing the hypotheses is shown in Figure 1.

## 3. Method

### 3.1. Procedure

The present study was conducted in Germany based on a cross-sectional survey supplemented by a qualitative follow-up. The quantitative data were gathered between June and September 2020. Between March and the early summer of 2020, Germany – as well as many other countries – experienced the first rise of COVID-19 infections and took various measures (e.g., physical distancing, lockdowns, working from home) to improve the situation (e.g., Zacher and Rudolph, 2022). The survey was conducted in a period where the incidence rates decreased for the first time directly after the Corona lockdown from March to May 2020 which was a suitable time period to gather data for a retrospective. At the end of the first survey, the participants were asked for their agreement to be contacted for an additional qualitative follow-up. This follow-up took place in November and December 2020 – considering a sufficient time lag with respect to the first survey in order to prevent any content-related interference (e.g., just repeating the item content of the quantitative survey within the free answers of the qualitative part). In autumn 2020, the second increase of COVID-19 infections began, so we could build on an enhanced awareness of a longer-term crisis.

Prior to collecting data, our study received ethical approval (affiliation of the second author; no. 2020-075).

### 3.2. Sample

#### 3.2.1. Quantitative survey

The sample was recruited via social networks and personal and professional contacts of the first author. The final sample consisted of 60 operational leaders. Operational leaders were identified by two filter questions. They were defined by direct staff responsibility and authority to give orders to their subordinate personnel as well as dependency of at least one hierarchical level within the organization, which is authorized to give orders to them. Regarding their hierarchical level, they labeled themselves for example as “division/department leader,” “team/group leader” or “branch manager.” Participants´ tenure in the organization and in a leading position was at least 6 months in order to ensure that they were able to compare working conditions before and during COVID-19 crisis. Most of the participants held a leadership position for a long time, with 32% even 10 years and longer. 20% of the participants´ organizational tenure was between 5 and 10 years and 48% 10 years and longer. The participants had between two and 123 followers (*M* = 14.84; *SD* = 18.22). The participants were from organizations of various industries (see Table 1) and worked in various corporate functions. 37% of the participants were female, and their age ranged from 25 to 68 years (*M* = 42.02; *SD* = 11.28).

**Table 1:** Overview of the economic activities of the participants’ organizations – Classification of Economic Activities, Edition 2008 (WZ 2008) (Federal Statistical Office Germany, 2008).

WZ 2008 code and description	Number of participants in the quantitative survey	Number of participants in the qualitative follow-up
C Manufacturing	10	4
G Wholesale and retail trade	2	
I Accommodation and food service activities	1	
J Information and communication	4	
K Financial and Insurance Activities	19	3
M Professional, scientific and technical activities	16	4
O Public administration and defence; Compulsory social security	3	3
Q Human health and social work activities	3	
Not specified	2	

#### 3.2.2. Qualitative follow-up

The qualitative follow-up was completed with 14 operational leaders out of the 60 participants from the quantitative survey. Of those, 36% reported to hold a leadership position for at least 5 years and 50% even 10 years and longer. 71% of the participants´ organizational tenure was 10 years and longer. The participants had between 5 and 12 followers (*M* = 8.31; *SD* = 2.10). The participants were from organizations of various industries (see Table 1) and worked in various corporate functions. 36% of the participants were female, and their age ranged from 30 to 68 years (*M* = 47.14; *SD* = 13.19).

### 3.3. Measures

#### 3.3.1. Quantitative survey

We used 5-point Likert answering scales to assess study constructs. Most of the items were adapted linguistically or complemented as regards content examples to the context of the crisis. If not stated otherwise the items began with the lead-in “Since the beginning of the COVID-19 situation …” or with a similar introduction in order to refer to the pandemic. All items are displayed in the Appendix (Supplementary material A). For each scale, an EFA confirmed a one factor solution.

##### 3.3.1.1. Work intensification

For the measure of work intensification we combined items from three different validated scales in order to cover those facets addressed by our research question. Two items were taken from Kubicek et al. (2015) measuring job demands in accelerated change with work intensification as a subdimension (exemplary item: “It is increasingly harder to take time for breaks”). As Kubicek et al. (2015) focus on quantitative work intensification, we supplemented three items of the Individual Job Impact scale by Caldwell et al. (2004), which gathers qualitative individual-level changes in job demands, expectations, and responsibilities in the course of organizational restructuring (exemplary item: “My job responsibilities have broadened”). Given that work intensification is closely related to work-privacy conflicts by affecting not just spare time during working hours but also private time we eventually included two items taken from the Copenhagen Psychosocial Questionnaire (COPSOQ) by Burr et al. (2019/exemplary item: “I am dealing with work matters more often outside my working hours”). The seven items of our combined scale showed a good internal consistency (*α* = 0.83).

##### 3.3.1.2. Emotional demands

To capture emotional demands we used three items taken from the COPSOQ (Burr et al., 2019/exemplary item: “I have to deal with my followers’ personal problems and worries”). We supplemented four items formulated by ourselves to tailor the scale to the specific context (exemplary item: “I have to communicate unpleasant decisions (e.g., with regards to job losses or substantial changes in my work unit”). The seven items showed a good internal consistency as well (*α* = 0.77).

##### 3.3.1.3. Organizational instrumental support

Regarding informational support, we used four items from the Informational Justice scale by Maier et al. (2007) and tailored them to the specific situation (exemplary item “The procedures within our organization in terms of the handling of the COVID-19 situation are reasonable”). Regarding practical support, we supplemented four items formulated by ourselves based on advice from the German Federal Ministry of Labor and Social Affairs (Federal Ministry of Labor and Social Affairs/BMAS, 2020/exemplary item: “Within our organization we have a central contact (e. g. a “crisis committee”) in terms of COVID-19 topics and questions”). The eight items showed a good internal consistency (*α* = 0.87).

##### 3.3.1.4. Occupational self-efficacy

The self-efficacy items were aimed to capture the participants´ general attitudes regardless of the crisis situation. We used the eight-item short version by Schyns and von Collani (2002/exemplary item: “When I am confronted with a problem in my job, I can usually find several solutions”). We supplemented them by two items of the C-Lead scale by Hadley et al. (2011) dealing with effective crisis leadership (exemplary item: “I can make decisions and recommendations even when I do not have as much information as I would like”). The ten items together also had a good internal consistency (α = 0.86).

##### 3.3.1.5. Irritation

Irritation was measured by four items of the scale developed and validated by Mohr et al. (2005/exemplary item: “I get irritated easily, although I do not want this to happen”). Internal consistency was good (*α* = 0.86).

##### 3.3.1.6. Exhaustion

Exhaustion was measured by three items of the Oldenburg Burnout Inventory by Demerouti et al. (2003/exemplary item: “During my work, I often feel emotionally drained”). This scale showed a good internal consistency as well (*α* = 0.91).

#### 3.3.2. Qualitative follow-up

The qualitative follow-up was conceptualized as a written online survey. The participants were asked to describe their work routine and their experiences with regard to work intensification and emotional demands. Concretely, they were asked to give at least one example how they experienced work intensification and emotional demands, respectively, during the first months of the COVID-19 crisis by describing and characterizing specific situations. To ensure a common understanding of the constructs, a short definition was provided. Furthermore, with regard to organizational support, the participants were asked to describe and evaluate the support they got from their employers categorized as helpful support, missing support and in terms of an overall judgment of the organization’s crisis management.

### 3.4. Data analysis

#### 3.4.1. Quantitative survey

To test our hypotheses, we conducted moderated hierarchical regression analyses using IBM SPSS Statistics 26. We first centered all variables around their mean scores and built interaction terms with the mean centered independent and moderator variables (compare Aiken et al., 1991).

The predicted two-way interaction effects were tested in four separate hierarchical analyses for the two predictors in combination with the two outcomes. The moderators were considered in parallel. In each hierarchical regression, the job demand was included in the first step of the regression equation, the two job resources in a second and the interaction terms in the third step. In other words, we examined the extent to which the interaction between job demands and job resources explained a unique proportion of the variance in the outcomes, after controlling for the main effects.

#### 3.4.2. Qualitative follow-up

Following qualitative content analysis (Mayring, 2010) we analyzed participants’ answers. This kind of analysis is based on a category system, which could be derived from a solely inductive or combined deductive and inductive procedure. In this case, we concentrated on an inductive procedure by developing categories out of the text material itself. Within the categories and the category description we summarized and abstracted the text material. For the labels of the categories, however, we have drawn on the relevant definitions and theoretical considerations with regards to work intensification, emotional demands, and organizational support, which can be considered a deductive element. The coding procedure was carried out by two independent coders – the first author and a research assistant. The first author defined the content analytical units and developed the categories from the individual statements of the participants and made her final assignment. The research assistant then repeated this assignment independently of the first author and provided feedback on the classification and the labels of the categories. Discrepancies were discussed and a common solution was determined. As an agreement measure for interrater reliability Cohen’s Kappa was calculated and interpreted according to Landis and Koch (1977).

## 4. Results

### 4.1. Quantitative survey

Table 2 depicts the means, standard deviations and intercorrelations of all study variables as well as the internal consistencies. Overall, work intensification and emotional demands yielded significant positive relations with negative mental health outcomes, supporting Hypotheses 1a and b and 2a and b.

**Table 2:** Means, standard deviations, internal consistencies, and correlations between the variables.

	Variable	*M*	*SD*	1	2	3	4	5	6
1	Work intensification	3.0	0.8	0.83					
2	Emotional demands	2.4	0.67	0.46 **	0.77				
3	Organizational instrumental support	4.1	0.7	−0.19	−0.27*	0.87			
4	Occupational self-efficacy	4.0	0.5	0.16	−0.18	0.43 **	0.86		
5	Irritation	3.0	0.9	0.42 **	0.48 **	−0.29 *	0.11	0.86	
6	Exhaustion	2.8	1.2	0.43 **	0.51 **	−0.30 *	−0.32 *	0.81 **	0.91

*N* = 60; Cronbach’s alphas are listed on the diagonal. * *p* < .05 ** *p* < .01

The correlations were in line with the results of our regression analyses (see Table 3 and Table 4). Work intensification was significantly and positively related to irritation (*b* = 0.47, *p* < 0.01) and to exhaustion (*b* = 0.61, *p* < 0.01) as well as emotional demands to irritation (*b* = 0.68, *p* < 0.01) and to exhaustion (*b* = 0.91, *p* < 0.01).

**Table 3:** Regression analyses work intensification.

	Irritation				Exhaustion			
*Direct effects:*	*∆R* ^2^	*b*	*SE*	*p*	*∆R* ^2^	*b*	*SE*	*p*
Work intensification		0.47	0.13	0.00		0.61	0.17	0.00
Organizational instrumental support	0.05	−0.21	0.17	0.22	0.15	−0.08	0.21	0.70
Occupational Self-Efficacy		−0.22	0.28	0.43		−0.99	0.33	0.01
*Moderation:*								
Work intensification ×								
Organizational instrumental support	0.08	−0.50	0.20	0.01	0.09	−0.66	0.23	0.01
Occupational Self-Efficacy		0.36	0.34	0.30		0.79	0.40	0.05
Total *R*^2^	0.26				0.37			

*b* unstandardized coefficient, *SE* standard error of *b*, *p* significance value. *N* = 60.

**Table 4:** Regression analyses emotional demands.

	Irritation				Exhaustion			
*Direct effects:*	*∆R* ^2^	*b*	*SE*	*p*	*∆R* ^2^	*b*	*SE*	*p*
Emotional demands		0.68	0.16	0.00		0.92	0.20	0.00
Organizational instrumental support	0.03	−0.25	0.17	0.14	0.06	−0.17	0.21	0.42
Occupational self-efficacy		0.10	0.27	0.70		−0.53	0.33	0.11
*Moderation:*								
Emotional demands^x^								
Organizational instrumental support	0.04	−0.24	0.21	0.25	0.02	−0.37	0.26	0.17
Occupational self-efficacy		−0.24	0.42	0.57		0.36	0.53	0.49
Total *R*^2^	0.24				0.28			

*b* unstandardized coefficient, *SE* standard error of *b*, *p* significance value. *N* = 60.

**Figure 2:**
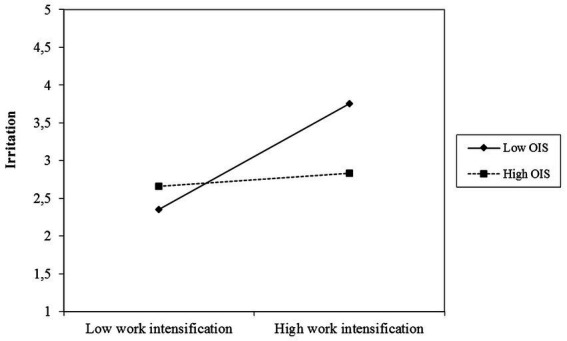
Moderation effect of organizational instrumental support on the work intensification – irritation relation.

Regarding Hypothesis 3, results indicated significant interaction effects for work intensification and organizational instrumental support on irritation (*b* = −0.50, *p* < 0.01) and exhaustion (*b* = −0.66, *p* < 0.01). Thus, Hypotheses 3a and b were supported. Figures 2 3 show that support effects the demand-outcome relation in the assumed direction.

**Figure 3:**
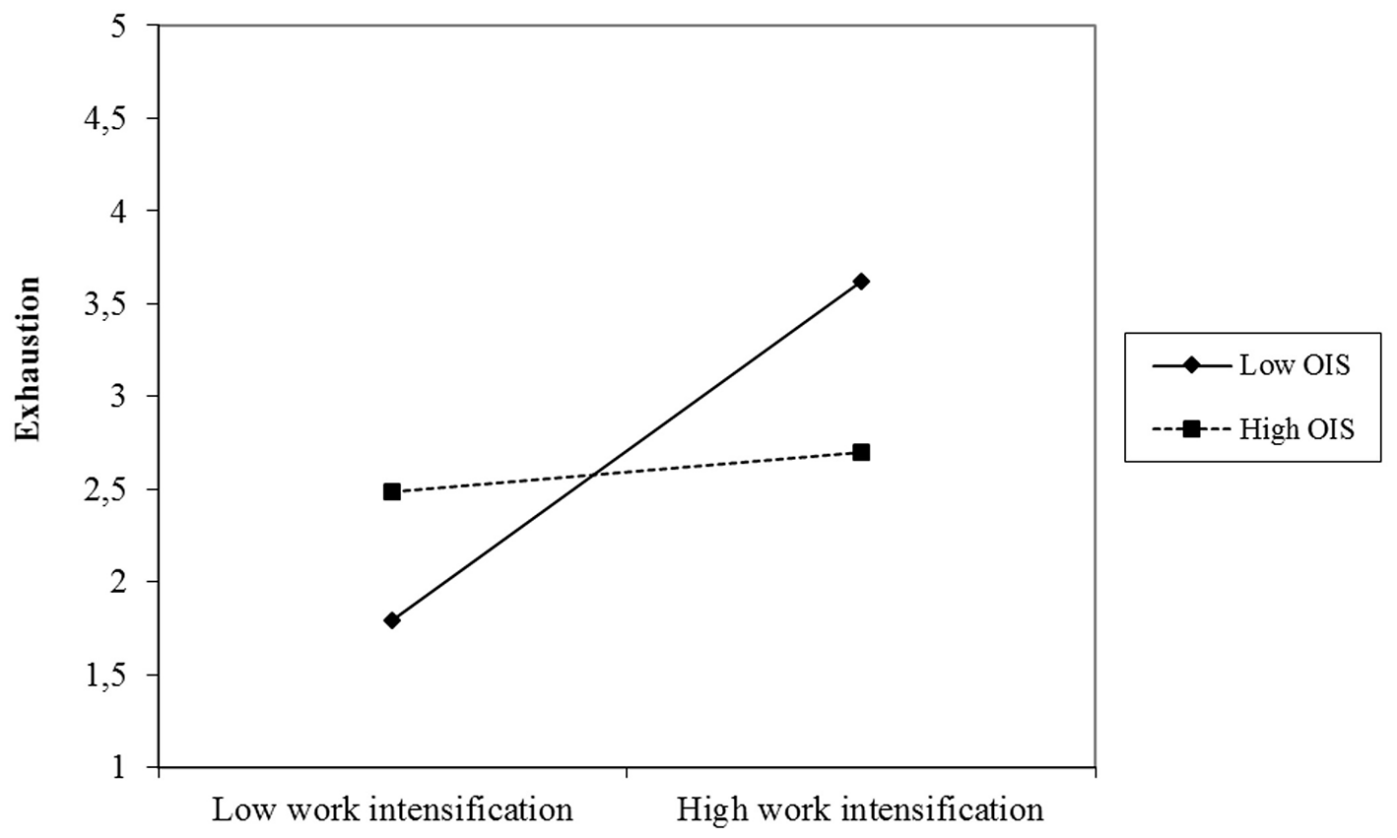
Moderation effect of organizational instrumental support on the work intensification – exhaustion relation.

**Figure 4:**
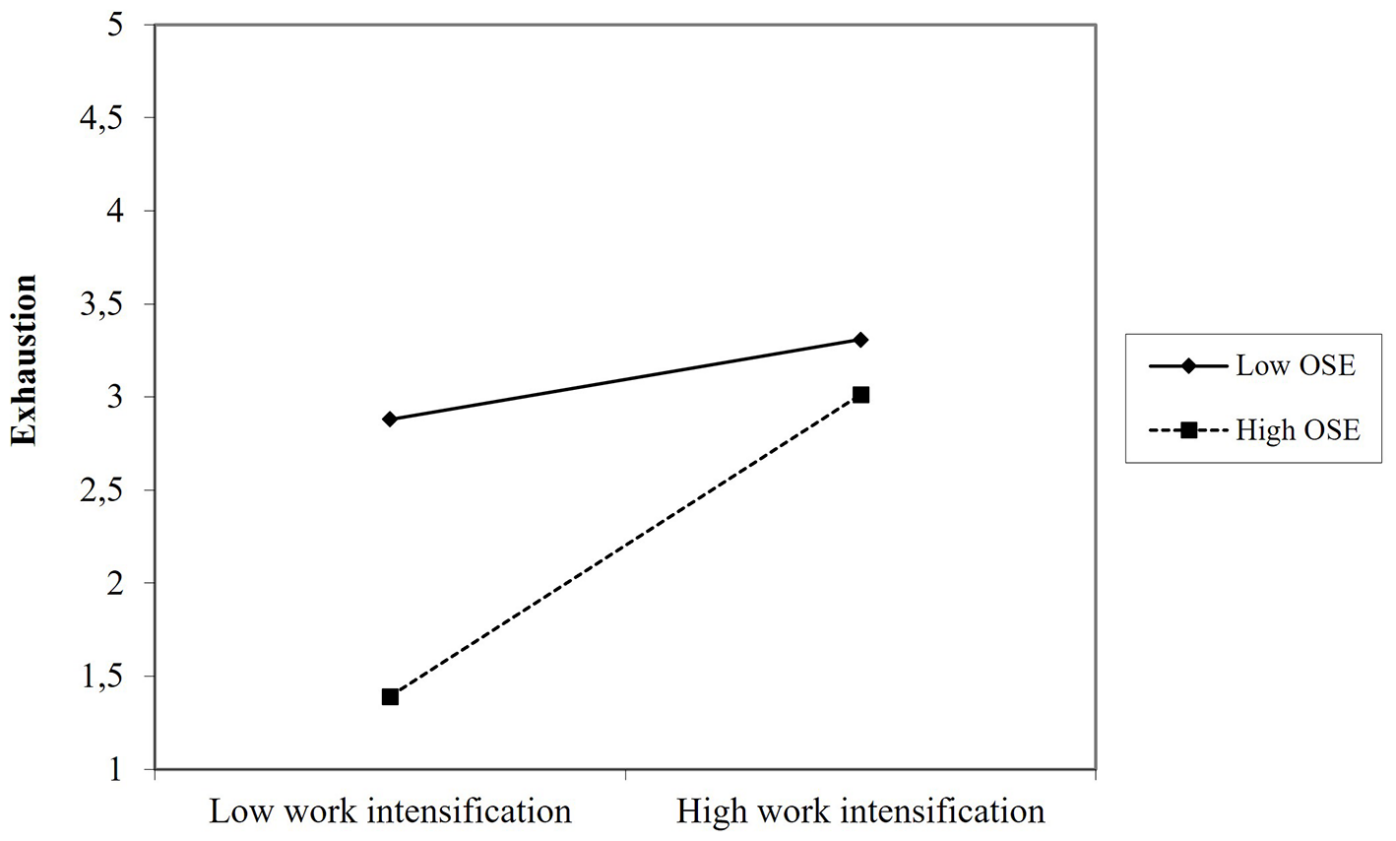
Moderation effect of organizational self-efficacy on the work intensification-exhaustion relation.

Work intensification and occupational self-efficacy show no significant interaction effect on irritation resulting in a rejection of Hypothesis 5 a. However, the interaction was significant for exhaustion (*b* = 0.79, *p* < 0.05) supporting Hypothesis 5 b. As depicted in Figure 4, the moderating effect of occupational self-efficacy contradicted the presumed effect, which will be considered in detail in the discussion section.

Hypotheses 4 and 6 had to be rejected due to non-significant interaction effects between emotional demands and the two resources.

### 4.2. Qualitative follow-up

The detailed results of the qualitative follow-up are summarized in Tables 5–7. For work intensification (see Table 5), we analyzed text material of 531 words in 29 statements/content analytical units. Regarding the interrater reliability the calculated Cohen’s Kappa of 0.73 showed a substantial agreement (Landis and Koch, 1977). We could find four categories in the participants’ statements. One category referred to *Work intensification associated with telework and virtual leadership/collaboration* (9 statements, 207 words) and consists of the two subdimensions *Transition phase and preparation* and *Working phase and collaboration* describing the expense of work due to organizing and living new forms of collaboration. The category *Work intensification due to emotional demands* (6 statements, 159 words) referred to those statements that link emotional challenges with work intensification describing them as demanding because they claim extra time from already scarce time resources. The two categories *General aspects of quantitative work intensification* (9 statements, 108 words) and *General aspects of qualitative work intensification* (5 statements, 57 words) reflected changes in the quantitative and qualitative nature of work equivalent to the construct definition given above.

**Table 5:** Summary of the results from the qualitative follow-up: work intensification.

Category	Content
Work intensification associated with telework and virtual leadership	*Transition phase and preparation* Analysis of and introduction into new software solutions for web conferences and new communication media (for oneself and the work unit); sometimes not functional and complicated Organization and assurance of functioning of work unit under bad technical conditions; availability of documents often just in an analogue way More time for preparation of web conferences than for normal meetings *Working phase and collaboration* Concentration of intense phases and less idle time on the one hand (“10 h web conference without breaks/physical exercise/ small talk”) and fragmentation on the other hand (more web calls and telephone conferences because they were easy to set up ➔ more disturbance of workflows; more communication channels)
Work intensification due to emotional demands	Time and effort needed for supporting employees, dealing with individual problems and finding individual solutions Necessity of compensation of less emotional ties between leader and followers, e.g., by more personal conversation with each follower ➔ high time invest
General aspects of quantitative work intensification	Higher amount of work and higher speed of work, less breaks Generally a higher need for planning, re-organizing, consultations under highly uncertain conditions
General aspects of qualitative work intensification	A feeling of being responsible for everything caused by a blurring of responsibilities; new responsibilities without more time resources and higher needs for decision making

**Table 6:** Summary of results from the qualitative follow-up: emotional demands.

Category	Content
Followers’ problems, worries, fears/Interface between work and private life	Own health status, health status of family and friends, risk of infection at the workplace (putting oneself and others at risk) Work-privacy conflicts (esp. in terms of child care) Social exclusion (esp. for people who live alone) Dealing with followers’ problems, worries, fears while partly being confronted with the same challenges themselves
Contradictory demands	Contradictions between … … attitude of organization’s management on the one hand [(no) sense of the seriousness of the situation] and the followers’ needs on the other hand at expectation of loyalty on all sides; this led to a take-over of organization’s responsibilities regarding crisis management due to a lack of clear rules … followers’ needs and the task requirements in the work unit (decreased performance due to responsibilities in private life; distribution of tasks), careful consideration of legitimate versus illegitimate concerns, difficult decision making
Socio-emotional (team) climate	Atmosphere of annoyance that could lead to overreactions on both sides; even more difficult in a virtual leadership setting due to loss of emotional ties Team conflicts (e.g.; due to a lack of technical equipment)

**Table 7:** Summary of results from the qualitative follow-up: organizational support.

Category	Content
Communication, information, orientation	Crisis contact/crisis committee, clear responsibilities Clear and transparent internal communication Consistent organization-wide measures, clear instructions on operating procedures Fast and unbureaucratic decisions ➔ As important prerequisites for leaders to pass on to their teams
Financial and practical support	Home office equipment Infection prevention materials Voluntary financial support from the organization, e.g., continued pay in case of suspected infection for employees who cannot telecommute ➔ Reduces conflict over limited technical resources and avoids presenteeism with high risk of infection
Flexibility of working time and space; Individual solutions	Home office, appropriate technical solutions Flexibility of working time and space as an opportunity to reduce work-privacy conflicts ➔ Individual solutions for all employees, including leaders themselves ➔ More decision latitude for leaders to find individual solutions for their teams
Participation and autonomy	Early and active involvement of leaders from all hierarchical levels in organizational decisions to enhance identification with crisis-related measures and to facilitate communication of unpleasant decisions Decisions latitude for operational leaders
Crisis as an opportunity for change	Returning to old structures too early Time was not used to test new forms of collaboration ➔ Strengthening the idea of “crisis as opportunity” by maintaining and building on positive developments
Trust	Trust (especially from higher levels) in leaders and on the issue of working from home
	➔
Prevention from performance pressure and role conflicts	Reduced target agreements, permission for postponing project deadlines Transparent prioritization of tasks ➔ Reduces work intensification for leaders
Encouragement of a positive (team) climate	Enhancing solidarity by organizational initiatives (e. g. voluntary “donation” of home office working hours to colleagues with responsibility for child care) Promoting team spirit, reliability and respect
Leadership training and development	Training regarding personal resources to strengthen resilience Technical training for leaders to gain competence to find suitable digital solutions for their teams

For emotional demands (see Table 6), we analyzed text material of 595 words in 19 statements /content analytical units. Regarding the interrater reliability the calculated Cohen’s Kappa of 0.83 showed an almost perfect agreement (Landis and Koch, 1977). We found three categories. The first category *Followers’ problems, worries, fears/Interface between work and private life* (12 statements, 184 words) summarized the diversity of challenges followers had to face and leaders had to deal with during the COVID-19 crisis. The second category *Contradictory demands* (4 statements, 236 words) described the multifaceted areas of tension leaders are confronted with. In addition, the third category *Socio-emotional (team) climate* (3 statements, 175 words) referred to an overall atmosphere that was created within teams during crisis.

In terms of organizational support (see Table 7) the participants should not only describe, but also evaluate the support they received and they wished to receive. Here, we analyzed text material of 631 words in 64 statements/content analytical units. Regarding the interrater reliability the calculated Cohen’s Kappa of 0.90 underlined an almost perfect agreement (Landis and Koch, 1977). We summarized the questions regarding the most helpful organizational support measures, the unavailable but desired support and the most critical factors, so that the table shows the important factors whose presence was helpful and absence was critical. We created eight categories, namely *Communication, information and orientation* (23 statements, 220 words); *Financial and practical support* (11 statements; 193 words); *Flexibility of working time and space/Individual solutions* (10 statement; 63 words); *Participation and autonomy* (4 statements, 50 words); *Crisis as an opportunity for change* (4 statements, 41 words); *Trust* (4 statements, 13 words); *Prevention from performance pressure* (3 statements, 21 words); *Encouragement of a positive (team) climate* (3 statements, 12 words) and *Leadership training and development* (2 statements, 18 words). In each category description we showed why these aspects are specifically important for our target group – leaders from the lower and middle management. In order to reflect the importance within our sample, the categories are ordered by number of statements and words.

## 5. Discussion

### 5.1. Integration of quantitative and qualitative results

Aim of this study was to deepen the understanding of leaders’ specific demands in the course of the pandemic and the role of personal and organizational resources in relation to their health. In line with our hypotheses, the results showed that work intensification and emotional demands are relevant regarding leaders’ health in crisis situations, such as the COVID-19 pandemic. Bespoke organizational instrumental support moderated the association between work intensification and mental illness.

Some results, however, were unexpected. First, low support seems to be more harmful than high support is helpful (see Figures 2, 3). This result underlines the importance of support and other organizational resources, but it also indicates that the helpfulness of resources can end in a saturation effect and that it is at least as important to reduce demands (see also Thomson et al., 2021). Second, for leaders with a high level of occupational self-efficacy, work intensification had a stronger positive association with exhaustion (see Figure 4). It is possible that leaders with a high level of occupational self-efficacy tend to invest more to be successful within demanding situations, which can result in even higher exhaustion. Against the background of autonomous self-endangerment, particular personal resources, may have exacerbating effects when combined with demands that can hardly be influenced. This is in line with the concept of personal demands, which assumes that resources can shift into a demand depending on the interaction with other work characteristics (Bakker and Demerouti, 2017). Third, we could find no buffering effect of the resources at hand for emotional demands. Possibly, emotional demands include conflicting and contradictory situations – this assumption becomes particularly apparent in the qualitative data – that are not addressed well enough by our resources. Apparently, the resources match work intensification much better than emotional demands and there may be other resources needed to buffer the health impairment due to emotional demands.

The qualitative part in our mixed method design served two purposes (Greene, 2008; Kuckartz, 2014). First, it enabled triangulation, as we could validate our scales due to a high overlap between the items and the descriptions and examples given by the participants (see, e.g., *General aspects of quantitative and qualitative work intensification* or the different categories regarding organizational support). Second, it ensured complementarity, as the qualitative data illustrate, expand and explain the quantitative results. This generated approaches for future research and concrete practical implications for leaders (see Practical implications). One example are the contradictory demands of different stakeholders leaders are confronted with – they are worth to have a closer look at. Furthermore, the qualitative results show that work intensification and emotional demands have strong associations. We are getting closer to the various and multifaceted reasons for the perception of work intensification and emotional demands. The significant interaction effect of work intensification and organizational instrumental support is likewise depicted in the qualitative data. Many aspects of our organizational instrumental support scale are assessed as beneficial factors by the participants and their absence might be an important predictor for a negative perception of work intensification. Finally, the importance of the organizational support category *Communication, information, orientation* is also underpinned by a look at the sample description of the qualitative sample. Although they are mostly experienced managers, these aspects were widely discussed by the leaders against the background of a crisis.

### 5.2. Limitations, strengths and implications for future research

Apart from the small sample size, the most obvious limitation is our single-source cross-sectional design. This means that common-method bias (Podsakoff et al., 2003) may have influenced the results and that we cannot draw causal conclusions about the directions of the effects. However, due to the mixed methods design we were able to better comprehend the effects and relations based on the qualitative answers of the participants, which, however, does not substitute a longitudinal or experimental design. Regarding the single-source design, it will be important to integrate the results of this study with those on crisis leadership and its consequences on followers’ well-being or to conduct new studies with a dyadic design (i.e., multisource data). As the introduction showed, our study’s objectives are closely connected to the question, which requirements should be fulfilled to enable good and healthy leadership. Especially resources play an important role for the question how much energy is still available for good and healthy leadership when facing multifaceted demands. It could be interesting to consider different leadership styles as dependent variables of working conditions and support structures of leaders. Transformational leadership (e.g., Bass and Avolio, 1995) with its high developmental character, task-oriented leadership with its crisis management function (e.g., Stoker et al., 2019) and health-oriented leadership (e.g., Franke et al., 2014) with its leaders’ self-care component are interesting target constructs. However, destructive leadership should also be considered as a possible result of unfavorable constellations of conditions and resource depletion of leaders (e.g., Burton et al., 2012). Besides the COR theory, related resource models and theories, e.g., with regards to resource allocation at the workplace (e.g., the theory of Selective Optimization with Compensation/Baltes and Dickson, 2001) are useful for a closer look at the association between leaders’ demands, resources, their health and their leadership behaviour.

Furthermore, the analysis of the interaction between personal and organizational resources should be deepened (Bakker and Demerouti, 2017), maybe by analyzing three-way interactions based on a more comprehensive sample. This could contribute to the question whether resources support each other (“fertile ground”-hypothesis) or compensate for the absence of the other resource. The most recent publications on COR theory (e.g., Hobfoll et al., 2018) address resource interactions (Resource Caravans) and the contextual requirements for using available resources in a suitable way (Resource Caravan Passageways).

### 5.3. Practical implications

To prevent mental health problems among leaders and more generally employees, organizations – concretely the upper management, Human Resource Management (HRM) and OSH practitioners – should provide resources, especially when it is difficult to reduce or redesign job demands (Bakker et al., 2005; Zacher and Rudolph, 2022). This is not only important in times of crisis, but for change in general as managing change is a new increasing task area for leaders even in small or medium-sized organizations (e.g., Ötting et al., 2021). As our study shows, extending work-related resources is important, but can have saturation effects, so that reducing work demands is at least as important (see also Thomson et al., 2021).

Based on the quantitative and qualitative results of this study, we recommend the following measures to improve and facilitate the situation of leaders in times of crisis and change, considering both behavioral and structural prevention and their mutual dependency:

- Give early and clear definition of leaders’ (additional) responsibilities and tasks within a new situation. Generally, communication, information and orientation are decisive in crisis and change contexts. Concurrently, it is important to ensure the possibility for individual decision-making.
- Involve leaders in important organizational decisions to enhance their level of identification with those decisions, but also to benefit from their expert knowledge regarding their everyday experience with their followers and operating procedures.
- Encourage lateral exchange among leaders of the same hierarchical level to reduce uncertainty and to support mutual learning.
- Emphasize leaders’ socio-emotional role: Establish employee support as part of the leader role (e.g., via target agreements), enable timely resources for supporting employees (e.g., by reducing manager-to-staff ratio) and train or coach them to constructively deal with emotionally demanding situations. In addition, supervision – as it is common in social professions – could be a suitable measure for leaders.
- As virtual leadership will stay an important part in the post-Covid time, integrate self-care for leaders in virtual leadership trainings to ensure work-privacy boundaries and detachment.
- Implement interventions to bolster leaders’ self-efficacy (Korman et al., 2022) as an important personal resource, but combine it with training for self-regulatory competencies to prevent leaders from exhaustion.

The consideration of psychosocial demands evoked by the COVID-19 pandemic has been explicitly stated by the recommendations of the German government (Federal Institute for Occupational Safety and Health/BAuA, 2020; Federal Ministry of Labor and Social Affairs/BMAS, 2020; Robelski et al., 2020). To integrate those demands and related resources in the risk assessment is not only considered in the Working Conditions Act, but also important in times of crisis, including all hierarchical levels. Following these recommendations organizations have a bespoke monitoring instrument, on which they can base their interventions when context changes.

## 6. Conclusion

This study underlines the necessity to consider leaders as a specific and “vulnerable” group affected by changes in general and the Covid-19 crisis in particular. We confirmed work intensification and emotional demands as important demands for leaders in the pandemic. Due to their hierarchical level, direct leaders and middle managers are dependent on bespoke organizational support. This support can buffer the health impairment risk of demands, while it is important that it matches those demands. Personal resources, such as self-efficacy, can play an ambiguous role. To sum it up, leaders are an important target group for HRM and OSH practitioners and should be supported with tailored interventions.

## Data availability statement

The raw data supporting the conclusions of this article will be made available by the authors, without undue reservation.

## Ethics statement

The studies involving human participants were reviewed and approved by ethics committee of Bielefeld University. The patients/participants provided their written informed consent to participate in this study.

## Author contributions

AW and GW contributed to the conception and design of the work, and revised it critically for important intellectual content. AW was responsible for the acquisition, analysis, and interpretation of data for the work, and drafted the work. All authors provided approval for publication of the content and agreed to be accountable for all aspects of the work in ensuring that questions related to the accuracy or integrity of any part of the work are appropriately investigated and resolved.

## Conflict of interest

The authors declare that the research was conducted in the absence of any commercial or financial relationships that could be construed as a potential conflict of interest.

## Publisher’s note

All claims expressed in this article are solely those of the authors and do not necessarily represent those of their affiliated organizations, or those of the publisher, the editors and the reviewers. Any product that may be evaluated in this article, or claim that may be made by its manufacturer, is not guaranteed or endorsed by the publisher.
