# Supplementary material for: Leaders’ mental health in times of crisis: work intensification, emotional demands and the moderating role of organizational support and self-efficacy
Source: Front Psychol. 2023 May 2;14:1122881. doi: 10.3389/fpsyg.2023.1122881 (PMC10186101; doi:10.3389/fpsyg.2023.1122881)
Supplement: Supplementary file 1 [file Data_Sheet_1.PDF]

Supplementary Material

Leaders' mental health in times of crisis: Work intensification, emotional demands and the moderating role of organizational support and self-efficacy

Anja Wittmers*, Günter W. Maier

*** Correspondence:** Corresponding Author: wittmers.anja@baua.bund.de

1 Appendix / Supplementary Material A – Items

Construct / variable	Instructions and items
Work intensification	Since the beginning of the COVID-19 situation …
	I am expected to do more work than I used to.
	I got new job responsibilities in addition to my actual tasks.
	I find greater demands placed on me.
	It is increasingly harder to take time for breaks.
	I am more often disturbed or interrupted in my work (e. g. by my followers' questions …).
	I am dealing with work matters more often outside my working hours.
	In my free time I am more often available for people I have to deal with at work.
Emotional demands	The following items refer to the time since the beginning of the changes caused by the COVID-19 situation.
	I have to deal with my followers' personal problems and worries.
	I have to support my followers with regards to their personal situation.
	I have to make unpleasant decisions (e. g. with regards to job losses or substantial changes in my work unit).
	I have to communicate unpleasant decisions (e. g. with regards to job losses or substantial changes in my work unit).
	My work is emotionally demanding.

	Towards my followers, I have to describe the situation within our organization differently from how it actually is.
	I have to hide my feeling in conversation with my followers.
Organizational Instrumental Support	Now we take a closer look on context and support your organization provides during these times. The following items refer to the time since the beginning of the changes caused by the COVID-19 situation.
	I feel well informed about important aspects and processes within our organization with regard the COVID-19 situation.
	Actual incidents and changes are communicated in a timely manner within our organization.
	If there is suspected or actual presence of infection, there are clear handling instructions and responsibilities.
	I have access to all information I need to fulfil my responsibility towards my followers with regard to occupational safety.
	Within our organization we have a central contact (e. g. a "crisis committee") in terms of COVID-19 topics and questions.
	The procedures within our organization in terms of the handling of the COVID19 situation is reasonable.
	If required, I have access to expert support/ information (e. g. in terms of occupational safety or technical questions).
	I could draw on reliable technical infrastructure (e. g. in terms of telework/ home office and digital communication).
Occupational Self-Efficacy	Please rate the following items with regard to your general attitude independently from the current crisis situation.
	When unexpected situations occur in my work, I know how to handle them.
	If I am in trouble at my work, I can usually think of something to do.
	I can remain calm when facing difficulties in my job because I can rely on my abilities.
	When I am confronted with a problem in my job, I can usually find several solutions.
	No matter what comes my way in my job, I'm usually able to handle it.
	I meet the goals that I set for myself in my job.
	I feel prepared to meet most of the demands in my job.
	My past experiences in my job have prepared me well for my occupational future.

	I can make decisions and recommendations even under extreme time pressure.
	I can make decisions and recommendations even when I don't have as much information as I would like.
Irritation	The following items refer to the time since the beginning of the changes caused by the COVID-19 situation.
	I have difficulty relaxing after work.
	Even at home I often think of my problems at work.
	I anger quickly.
	I get irritated easily, although I don’t want this to happen.
Exhaustion	After work, I tend to need more time than in the past in order to relax and feel better.
	During my work, I often feel emotionally drained.
	After my work, I usually feel worn out and weary.
